# Exploring the Effectiveness and Sustainability of Trust Based Relational Intervention (TBRI®) as a Trauma-informed Approach in Two Tasmanian Child and Family Learning Centres

**DOI:** 10.1007/s40653-023-00574-6

**Published:** 2023-10-03

**Authors:** Elspeth Stephenson, Helen Yost

**Affiliations:** School of Education, University of Tasmania, Launceston, TAS Australia

**Keywords:** Early years, Adversity, Educator learning, Trauma approaches

## Abstract

The impact of adverse childhood experiences (ACEs) is well documented and poses challenges for all those living and working with children who have experienced early adversity. The need to be trauma-informed when working with children in all educational settings is now recognised as essential if healing and learning are to take place. There are an increasing number of trauma-informed approaches available, but empirical evidence that supports their efficacy, particularly in the early years of education, is currently scarce. This paper presents the findings of a small-scale study which explored early childhood professionals’ perceptions of the effectiveness and sustainability of one trauma-informed approach, Trust Based Relational Intervention (TBRI®). Used widely across the US and Europe, TBRI® is relatively new to Australia and was trialled for the first time in this Tasmanian study. Substance Abuse and Mental Health Services Administration’s (SAMHSA, 2014) concept of trauma and guidance for a trauma-informed approach was used to provide a theoretical framework. Online surveys were used to gather data after each professional learning workshop and three and six-months later. Participants reported several positives of TBRI®, including self-development and improved outcomes for children. Whilst challenges/barriers to using the approach were noted, many related to contextual issues rather than to TBRI® specifically. Findings also showed that equipping families with a similar skill set would be advantageous and integral to effecting sustainable change.

## Introduction

Experiences in the first years of life will set a path that has the potential to influence life trajectory (Cozolino, 2014; Purvis et al., 2013; Teicher & Samson, 2016). When these experiences are positive, consistent, and nurturing, shared with caring and loving adults, a child will begin to grow and thrive physically and emotionally but when experiences are inconsistent, negative and/or abusive, autonomic defence mechanisms work to enable survival (Cozolino, 2014; Porges, 2017). This situation can have a profound impact on the developing brain (Cozolino, 2014; Teicher et al., 2003). Whilst adaptive brain development created by adverse experiences is essential for survival in an unsafe environment, such adaptations do not support the individual to function where the need for survival is no longer necessary. In a seemingly safe and supportive environment, such as early childhood education and care or school, these adaptations can result in challenging or inexplicable behaviours that can be frightening and often dangerous. Exhibitions of such behaviours can leave educators struggling to cope which can erode teacher efficacy, particularly when previously effective educational practices are unsuccessful or escalate a problem situation. Here the need to be trauma informed in practice is essential if children are to flourish and educators are to maintain a sense of wellbeing and efficacy in their role.

Childhood trauma has been identified as “America’s hidden health crisis” (ACEs Connection, 2016 as cited in Thomas et al., 2019, p. 43), and described by van der Kolk (personal communication, August 2, 2018) as at global epidemic proportions and growing exponentially. Thus, the numbers of children in educational settings with trauma histories is increasing; a situation that is likely to continue (Oberg, 2022; Stipp & Kilpatrick, 2021) and poses a very real challenge for teachers, not only in terms of their practice but also their wellbeing (Alisic et al., 2012; Berger et al., 2020; Oberg, 2022; Souers, 2018; Stipp & Kilpatrick, 2021; Thomas et al., 2019).

The need to be trauma-informed is now widely recognised (Alisic, 2012; Alisic et al., 2012; Bath, 2015; Mendelson et al., 2015; Substance Abuse and Mental Health Services Administration [SAMHSA], 2014), having clarity around what this means and how to achieve this status, particularly in education, can be perplexing.

Understanding the impact of adverse experiences on a child’s growth and development is an essential starting point for educators, as it promotes a trauma-informed approach to practice that can help children get back to the path of healthy growth and development (Bath, 2008). According to SAMHSA (2014), however, understanding alone, “is not sufficient to optimise outcomes for trauma survivors nor to influence how service systems conduct their business” (p. 9). The significance of context, and those working within that context also needs to be recognised. Bath (2015) concurred with this concept and looked more closely at what happens in the *other 23 h* when children are in environments other than therapy and in the care of non-clinicians. Here he identified three critical aspects of practice that are “fundamental and universal” across all trauma literature and enacted in trauma informed environments (p. 18). These are: the development of safety, building connections through healing relationships, and growth of self-regulation and coping skills. Whilst depicted as three distinct pillars, Bath noted that safety, connections, and coping were closely inter-related. Felt safety can only be achieved in the presence of positive connections, which in turn supports coping and self-regulation (Bath, 2015). These essential aspects of a trauma-informed environment “provide a roadmap for success with children…exposed to chronic adversity” (Bath, 2015, p. 10), choosing a trauma informed approach that enables educators to develop skills to navigate such a path consistently within an organisation can still be a challenge and identified as “overwhelming to educators” (Thomas et al., 2019, p. 445).

Whilst there are an increasing number of studies exploring the effectiveness of trauma-informed approaches available to educators (Dorado et al., 2016; Holmes et al., 2015; Parris et al., 2015; Shamblin et al., 2016; Stokes & Turnbull, 2016), these have largely been carried out in primary and secondary school settings and mostly conducted in the US. Very little research has been done to explore approaches relevant to the Australian context or conducted in the birth to age five setting. Furthermore, research to explore trauma informed practices in schools has been conducted from various disciplines but educators are “underexamined” (Thomas et al., 2019). The study reported in this paper was conducted in Tasmania across two Child and Family Learning Centres (CFLCs) which cater for families with children aged birth − 5 years. The aim of this study was to explore the effectiveness of one trauma informed approach, Trust Based Relational Intervention (TBRI®), particularly from the perspective of the early childhood professional. TBRI® is relatively new to Australia and was trialled for the first time in an education and care context in this study. TBRI® is discussed in more detail below.

### Trauma-informed Approaches to Practice

There are now many resources available to support educators to become trauma-informed in their practice including, academic and professional articles, guides, toolkits, and advice on best practice. These can be found on most department of education websites internationally or from more specific trauma-based organisations such as The National Child Traumatic Stress Network in the US or Australian Childhood Foundation in Australia. Yet, as noted above, empirical evidence to support efficacy of the various programs and resources offered is still scarce (Thomas et al., 2019). From their explorations, Thomas et al. (2019) found that “the emergence and rapid growth of trauma-informed care into the educational realm… has occurred with no standard, formally agreed upon terms or framework when it comes to implementing trauma-informed practices in…schools” (p. 441). This reflects the need for a stronger evidence base (Chafouleas et al., 2016) that may guide early childhood professionals and school-based educators to make informed decisions about the path they choose to becoming trauma-informed.

Research undertaken to date includes evaluations of programs such as the Heart of Teaching and Learning (HTL) (Day et al., 2015), the HEARTS (Healthy Environments and Response to Trauma in Schools) program (Dorado et al., 2016), the Head Start Trauma Start (HSTS) (Holmes et al., 2015) and Trust Based Relational Intervention (TBRI®) (Ausley, 2022; Parris et al., 2015; Stipp & Kilpatrick, 2021). Each study was quite different in approach and carried out in differing contexts ranging from residential high schools through to early childhood community settings. Some approaches included intensive therapy for children (Dorado et al., 2016; Holmes et al., 2015), whilst others included no individualised therapy at all (TBRI®). Also, children needed to be referred for some programs (HSTS) (Holmes et al., 2015), whereas other programs had a school wide focus (Day et al., 2015). This, along with varying methodological approaches was reflected in the disparity of findings, although some similarities were reported. Each model shared the common element of professional learning for all staff. Although implementation of this learning was varied in approach and duration, it was seen to be a “change catalyst, central to becoming trauma informed and improving motivation” (Avery et al., 2021, p. 392). All studies identified an increase in teacher knowledge, which could be an expected outcome of any professional learning.

Shamblin et al. (2016) in their study exploring a community partnership approach to becoming trauma-informed, noted that in addition to increased knowledge, teacher confidence and hopefulness for change for children and families was an important finding. This finding was reflected by Reid et al. (2018). All studies reported an increase in student attention and ability to access learning. Decreased negative externalising behaviours were also a common finding, with Parris et al. (2015) noting a 93.5% decrease in negative behaviours school wide after two years of implementation. These findings suggest that, despite many limitations identified, all of these interventions were effective in improving outcomes for children. The efficacy of each model in terms of context was not discussed, but is also an important consideration, since the complexity and individuality of a school system, along with the need for cultural responsiveness (SAMHSA, 2014) should not be overlooked.

Zakszeski et al. (2017) found that interventions in the reviews they explored were mostly implemented by external clinicians, with specific populations, and mostly used rating scales to assess psychopathological symptoms. These parameters are potentially problematic, since educators do not have clinical expertise as part of their educator toolkit (Perry et al., 2016). Similarly, clinicians do not necessarily have the appreciation of what is required to lead a busy educational space; an understanding helpful if an approach is to have success in an educational context (Thomas et al., 2019). In addition, the role of the educator was brought into question, where the lines between healing and learning became blurred (Oberg, 2022).

Studies undertaken specifically in ‘everyday’ schools where teachers’ perspectives on the effectiveness of an approach were sought are rare. Yet the need for teacher perspectives is an important one since successful reforms are aligned with educator values and beliefs (Cohen & Mehta, 2017). Beliefs drive practice and being cognisant of beliefs allows for reflection in action (Schon, 2016) which in turn creates practice change (Door, 2014).

Stipp and Kilpatrick (2021) gathered participant perspectives whilst exploring the effectiveness of TBRI® in a school setting with students aged 5–12 years. The primary finding from this study was that TBRI® professional development was both “socially acceptable and valuable for participants” (p. 77). Specific findings suggested that the teaching of individual topics relevant to participants’ everyday teaching was most helpful, particularly when they could be related to “real classroom life” (p. 75). Similarly, implementation strategies were identified as being of most benefit to their learning, along with the opportunities to discuss these with colleagues. Whilst changes to practice were not overtly measured in this study, participant responses indicated that specific tools and practical techniques were beneficial and had changed their practice (p. 77). These findings were echoed by Reid et al. (2018) who sought principal teachers voices in response to a school-wide TBRI® implementation program. Environment change was also identified here, with one principal noting that “when the students feel loved, safe, and successful, learning can take place” (p. 12). Professional learning in the TBRI® approach was identified to have increased teacher confidence and capacity to create such an environment. Thus, the voice of the teacher proved to be an important component in understanding how practice change could be supported. Here, the need to support the development of the craft of teaching to promote informed decisions about strategy choice is important if practice change is to occur, particularly considering that a paradigm shift from *doing* trauma-informed to *being* trauma-informed is necessary (Avery et al., 2022; Stephenson, 2023).

### Trust Based Relational Intervention (TBRI®)

TBRI® is an approach to working with children and young people who have experienced adversity. This approach requires no clinical training and has a focus on meeting the needs of educators so that they can meet the needs of all children in their care to support their learning. As such, it does not have a focus on any specific populations but is designed to support the needs of everyone within a community. When TBRI® is the underlying approach used within a setting, meeting the needs of everyone in a safe and compassionate environment is the primary goal (Reid et al., 2018).

Emerging from lived experience and grounded in theory, TBRI® was identified by Avery et al. (2021) as one of only four school-wide trauma-informed approaches globally that met at least two of the three essential elements of trauma-informed systems, and all six key principles of trauma-informed care (as defined by SAMHSA (2014).

TBRI® is a supportive guide to building understanding of the impact of trauma, identified above as a critical element of any trauma-informed approach. It compels those learning the approach to challenge personally held beliefs about themselves, children, and their development. In so doing, it deepens the practitioner’s sense of understanding and builds compassion. As a holistic intervention, TBRI® acknowledges and explains seemingly inexplicable behaviours, supports the recognition of symptoms of early trauma and provides strategies and tools for practitioners to apply this knowledge and understanding to their practice. In so doing, individual needs can be identified and met, development and healing can occur, and learning can be supported. Attachment theory forms the basis of TBRI®, with relationships being central to the approach and its effectiveness. Three Principles; Connecting, Empowering, and Correcting provide an overarching framework for the approach, with each principle having strategies to support implementation. TBRI® has been adopted in over 30 countries and implemented within various contexts including family homes, educational sites, orphanages, juvenile detention centres and community-based programs. As an approach to supporting trauma-informed practice, TBRI® is said to be accessible and effective for all who live and work with children (Purvis et al., 2013). Despite having an empirical evidence base in the US (Call et al., 2014; Parris et al., 2014; Reid et al., 2018; Stipp & Kilpatrick, 2021) the effectiveness of TBRI® as a trauma-informed approach is yet to be documented in Australia, and more specifically in the early years of education. Thus, the researchers were keen to find out how early childhood professionals working in Australia would respond to the approach and whether they would find it of use to them in their everyday practice when working specifically with young children, particularly those who were experiencing or had experienced adversity. In addition, the researchers were also interested to see whether the approach, if adopted, would remain in these professionals’ practice over time.

### Theoretical Framework

SAMHSA’s (2014) concept of trauma and guidance for a trauma-informed approach was used to provide a theoretical framework to understand the perceptions of participants in relation to the effectiveness of TBRI® in their workplace. This concept is grounded in four assumptions and six principles that are “key [to a] trauma informed approach” (p. 9). The key assumptions are called the four Rs. These refer to the *realisation* of how widespread trauma is, including understanding the effects of having a trauma history, how all people within a context *recognise* signs and symptoms of trauma, how the program *respond*s by “applying the principles of a trauma informed approach to all areas of functioning” (SAMHSA, 2014, p. 10), and finally how the approach *resists* doing further harm to anyone within the context.

The six principles which are non-specific and intended to be generalisable across contexts, include: Safety, both physical and psychological; Trustworthiness and Transparency, where all interactions are transparent and building trust is the aim; Peer Support, where connection with caregivers and others who have trauma histories is seen as an important aspect of healing and recovery; Collaboration and Mutuality, which recognises that healing happens in relationships and where power is shared; Empowerment, voice and choice, which recognises the loss of voice for those with trauma histories and seeks to re-establish a sense of empowerment through decision making and efficacy; and finally, Cultural, Historical, and Gender Issues, which honours cultural connections by incorporating policies and processes that reflect the racial, ethnic and cultural needs of individuals within a context, whilst addressing intergenerational trauma (SAMHSA, 2014).

As noted earlier, Avery et al. (2020) identified TBRI® as meeting all six SAMHSA (2014) principles. These are embedded throughout the TBRI® approach. The TBRI® Connecting principle and related strategies embody all six SAMHSA principles. The TBRI® Empowering principle and related strategies align closely with principle five, and the TBRI® Correcting principle and strategies respond directly to principles four and five. Whilst these principles could clearly be aligned to TBRI® in theory, the four Rs were used more specifically as a lens through which to view participant responses in relation to the effectiveness of TBRI® in practice. The research questions that informed the study were:

1. What are participants’ perceptions of TBRI®?
2. How effective was TBRI® as a trauma-informed approach within the context of a Tasmanian CFLC?
3. How sustainable were changes to practice over time?

### Context of the Study

This qualitative study was undertaken in two Tasmanian Child and Family Learning Centres (CFLCs). Tasmania is a small island state, situated approximately 240 km south of mainland Australia with a current population of just over five hundred thousand. Tasmania has a high percentage of children who have experienced early adversity (Commissioner for Children and Young People, 2018). Thus, support for all those who work with children who have experienced adversity has become a priority.

In Tasmania, CFLCs have been established in low socio-economic communities to improve the health and wellbeing, education and care of children aged from birth – 5 years (Taylor et al., 2017). The centres provide families with a single point of entry to various services, including health care, education and early development, nutrition, and parenting assistance. To respond to the needs of families, their staff includes allied health and education professionals. As such, CFLCs were identified as ideal settings for this study, since working with vulnerable children and families is a daily experience for professionals working in this context and the need to be trauma-informed in their practice a necessity. Two CFLCs agreed to take part in this study. The 29 participants comprised of early childhood educators (n = 22), site nurse (n = 1), psychologist (n = 1), speech therapist (n = 1), and library technicians (n = 4). Across both centres respondents were predominately female (n = 27), with two males located in one site.

## Method

Once ethical approval was obtained from the University of Tasmania’s Human Research Ethics Committee, the two CFLCs were enlisted for the study. The eight-hour TBRI® caregiver package was delivered across three professional learning workshops at each centre by a TBRI® qualified practitioner. Attendance at these workshops was employer mandated, however participant involvement in the research project was entirely voluntary. Prior to starting the first workshop, all participants were given an information sheet outlining the study, invitation to join, and time to ask clarifying questions.

A Qualtrics survey was used at the end of each professional learning workshop, and at three and six-months post workshop delivery to gather data. This was accessed via a hyperlink, emailed to each participant by a research assistant. Participant email addresses were provided by each centre manager. Completion and submission of the Qualtrics survey was deemed to be informed consent to participate in the research project. To ensure participant anonymity and enable the researchers to cross reference data collected over time, responders answered three questions, and in doing so, created their unique alpha/numerical identification code. Pseudonyms were given to participating centres when reporting the data.

The post workshop surveys sought participant perceptions of the effectiveness of TBRI® as an approach to supporting trauma-informed practice. Here participants were asked to identify potential positives and challenges/barriers to implementing TBRI®. Since the workshops were spread over several weeks, participants were able to trial new skills in their workplace and report back.

At three- and six-months post workshops, the survey questions sought to explore the sustainability of the approach over time. Here participants were asked to share whether they were still implementing the approach and if so, how it was affecting their practice.

The data were analysed by both researchers using a constant comparison and inductive approach (Patton, 2002). An initial review of the data was carried out followed by open coding of key sentences/phrases. Reading and re-reading participant responses identified patterns. This enabled common phrases to be classified and coded with possible schemes tested for *best fit*. This process was carried out individually by each researcher to increase reliability, then results were compared (Patton, 2002). These results were combined with multiple themes being identified. The themes were then condensed and recoded.

Once inductive thematic analysis had been completed, deductive analysis was undertaken using SAMHSA’s (2014) four Rs. This process enabled the researchers to ‘affirm and authenticate’ the suitability of the inductive analysis (Patton, 2002, p. 454) in relation to the effectiveness of TBRI® as an approach to trauma-informed practice.

## Findings and Discussion

The findings reporting participants’ perceptions of the positives and challenges/barriers of TBRI® as a trauma-informed approach (RQ1) are shared below. The interpretation of these findings from a theoretical perspective (SAMHSA, 2014), (RQ2) are discussed. Data pertaining to the sustainability of changes to practice (RQ3) are also presented.

### Participants’ Perceptions of the Positives of TBRI ®

Participant responses to the positives of TBRI® revealed four strong themes: self-development, relationships, improved outcomes, and the evidence-base of the approach.

#### Self-development

The most common theme that arose from participants’ perceptions of the positives of TBRI® was self-development with more than half of participant comments falling within this theme. Three sub-themes within self-development were identified, (i) new knowledge, about topics such as attachment, early brain development, understanding the impact of trauma, and the *realisation* of the extent of childhood trauma, (ii) strategies gained, and (iii) changing practice.

In the first sub-theme, new knowledge, one participant stated, “it fits so well with my existing attachment knowledge - it is like an extension”. Another noted “I have gained a greater understanding of what trauma can do to a person’s psyche. This will help me personally give empathy for our families that are travelling this road”. Another participant recalled their new knowledge as “understanding that trauma has lifelong implications – adult behaviours and intergenerational issues are huge in our…space” and another “understanding… offers the practitioner an alternative way of seeing children who don’t necessarily ‘fit’ in societies mainstream rules about what is so-called ‘normal or acceptable’ in terms of behaviour”. In *realising* the extent of trauma, one participant reflected on new knowledge and stated “[TBRI®] presents a coherent and comprehensive explanation of the extent of trauma damage” whilst another commented “it is good to understand that no matter how old people are, we may never truly know the triggers and that we should never jump to conclusions”. These comments highlight how the new knowledge gained during the professional learning resonated with participants as it extended their prior knowledge, was relevant to their current working context and they could see how it would support their practice.

The second sub-theme identified was strategies gained. Here participants highlighted how new strategies developed from the TBRI® workshops had supported them to *recognise* the signs and symptoms of trauma and how to *respond* more appropriately to children. One participant shared how “[TBRI®] has taught me to observe children even more to get good insight into remembering they may be from hard places…[it] provides tools for connecting with children”. Another participant commented on connecting strategies, stating that “this intervention helps me gain insight into how I can connect with children and build trust and a relationship” and another “I’m beginning to really understand how to better communicate with children … I’ve put what I’ve learned into practice … and have gained very rewarding results”.

The quality of inter-personal connection is key here since “it is only in relationships with others that a child can begin to feel safe” (Bath, 2015, p. 7). Effective communication with a child, particularly non-verbal communication and voice intonation, is critical to support the development of felt safety, since the body looks for ‘cues’ of safety which are often found in non-verbal communication (Porges, 2017). Felt safety is a “core developmental need of children” (Bath, 2015, p. 6) and is recognised as an essential state for social engagement, healing and learning (Dana, 2018). Knowing how to connect, therefore, is recognised as core to successful outcomes when working with children with trauma histories (Bath, 2008; Porges, 2017; Purvis et al., 2013).

The third sub-theme identified was changing practice. Here participants reported that new knowledge enabled them to reflect on and change their practice by employing the strategies taught. This was particularly apparent when referring to challenging behaviours. This point is highlighted in the following quotes: “[I am] no longer seeing behaviours as being difficult or unacceptable”, another noted that TBRI® gives “a sense of how to deal with children who have trauma impacting their life and how to deal with the behaviours that come with these children”. Further, “TBRI[®] …explains that behaviours have a need behind them”. The ability to see behaviour as a communication tool is critical to how professionals respond. When a practitioner listens to a behaviour and meets the need being expressed, the reduction of unwanted behaviour and a building of trust is a likely outcome. Conversely, if a behaviour is seen as wilful disobedience, a punitive response may result, which at best will escalate the behaviour and at worst cause re-traumatisation (Purvis et al., 2013). Listening and responding to a child’s needs is fundamental to the development of safety, healing and learning and ultimately being trauma-informed in practice.

#### Relationships

The second theme that arose from participants’ perceptions of the positives of TBRI® was relationships. Here two sub-themes were identified, (i) centrality to the TBRI® approach, and (ii) building trust.

In sub-theme one, participants were reassured by the centrality of relationships to the TBRI® approach. This was highlighted in the response from one participant who wrote that a positive of TBRI® was “the focus and value it places on relationships”, whilst another noted “TBRI [®] is important due to many people not thinking about the relationship formed with children and adults and this puts it in the lime-light”. Other participants shared these views, stating “TBRI [®] …allows the practitioner to build deep connections with the child” and “it highlights how everyone’s interaction can have a positive impact on a person no matter how seemingly small”. The identification of the significance of relationships demonstrates participants’ understanding of how to *respond* to children in a trauma-informed way.

In the second sub-theme, building trust, participants appreciated the strategies offered, noting that TBRI® had taught them how to: “build trust within a relationship to ensure that the relationship can develop in a positive way”; “meet the child’s needs through relationships”, and “focus on building relationships before looking for strategies for corrections”. Another participant stated that through TBRI® they had learnt “how to connect with children, get them engaged and build a relationship of trust”, whilst another stated “[TBRI®] helps me to gain insight into how I can …build trust in a relationship and give the right care”. Evidently, participants perceived that TBRI® strategies would support relationship building. This is an important finding since relationships are critical for connection (Bath, 2008), supporting felt safety (Porges, 2017) and are said to be the “main mode through which children develop” (Hayes et al., 2017, p. 30).

#### Improved Outcomes

Improved outcomes, the third theme, emerged as a positive of TBRI®. Here, two sub-themes were identified, (i) supporting vulnerable children and families, and (ii) supporting whole communities.

In the first sub-theme, participants reported that TBRI® would enable them to support improved outcomes for both children and their families. One participant identified a positive as “[it can] support children and families build strategies and skills to support their way forward positively” whilst another noted that “you can help a child or family have a better life”. Another participant stated, “learning about TBRI® has a very positive aspect so that we can help children now and into the future to have happier healthier lives”. After trialling new strategies, one participant wrote “the positive aspects are the significant changes in these little people, how they learn to respond, how they interact and how they learn to feel safe” whilst another noted “working in this way really does help families”. These comments show participants’ understanding of what is required to *respond* in a trauma-informed way.

The idea that TBRI® is a way of working to improve outcomes for all community members, rather than just for a specific cohort was highlighted as the second sub-theme. Here participants noted that “you can use the strategies in everyday life, with children who don’t come from hard places, and also those who do” and, “adopting the strategies used through TBRI® can be beneficial for all children”. Finally, the notion that TBRI® has the potential to be a transmissible approach for professionals to support improved outcomes was raised. Here participants stated that TBRI® could be useful “for staff across various roles” and reported that “this framework provides an excellent way for providing shared language and ways of working”. Consistency of an approach across all those who touch the life of a child is an important finding since it is considered integral to successful outcomes for vulnerable children and families (Coates, 2017).

#### Evidence-based Approach

The final theme that arose from participants’ perceptions of the positives of TBRI® was its evidence-based approach. This theme related to the knowledge that TBRI® had an evidence base and was built on theoretical concepts that directly aligned with participants’ knowledge and work. Participants’ comments showed a sense of reassurance that TBRI® was based on evidence. One participant stated that “[there is] lots of information around about trauma but this is the first program I have heard of which deals directly with this that is research based” whilst another noted that a positive was “the evidence base…links directly to my work…and so underpins all we do” and lastly “there is no doubt in my mind that the evidence and research base [is] an absolute in supporting and engaging with all children.” These views further demonstrate a confidence in the approach that supports practitioners to *respond* appropriately, *realise* the effects of having a trauma history and *resist* unintentional re-traumatisation.

Finally, participant perceptions of the positives of TBRI® can be summed by the following quotes:

I love the way it considers everything - meaning the person as a whole from in utero to now [and] there is something practical that we can do…that change or improvement can happen, and we can empower our little people [and lastly, TBRI®] presents a coherent, comprehensive explanation of trauma damage, which then leads to targeted interventions that make sense and have a good rationale for use.

### Participants’ Perceptions of the Challenges/barriers of TBRI®

Participant responses to the challenges/barriers of TBRI® also revealed four strong themes: personal and professional capacity, time, continuity of approach, and intergenerational trauma.

#### Personal and Professional Capacity

The most common theme that arose from participants’ perceptions of the challenges/barriers of TBRI® related to their personal and professional capacity. Here four sub-themes were identified, (i) efficacy, (ii) enacting TBRI® principles, (iii) being mindful in practice, and (iv) changing beliefs.

In sub-theme one, participants wrote about their own sense of efficacy and being able to put the processes of TBRI® into practice. This included being able to trust themselves to enact the principles of the approach whilst having the confidence to do so. One participant wrote that for them “trusting yourself and your personal and professional skills to support another through a challenging time” was a challenge, whilst another noted a challenge to be “working out the strategies that the individual child needs to be able to find their voice”. Here this participant has *recognised* the loss of voice as a potential symptom of trauma. Another participant identified that “having the confidence to read the moment to support the child” was a challenge for them. This suggests participants understood what was required but lacked the confidence to *respond* in practice.

The second sub-theme, enacting TBRI® principles, saw some participants identify certain aspects of the approach as more challenging than others. Practising the correcting principles and associated strategies were particularly noted. These principles focus on behaviour and rely on a caregivers’ nurturing through relationship while providing structure to enable the child to develop skills they need to be successful. Here the balance between structure and nurture is important to ensure the child feels safe but is also developing. During the professional learning, through a process of self-reflection, participants explored these aspects in relation to their own practice. For some the structure element was identified as a challenge as articulated by this participant “I wish to focus on learning to move from an over-protecting style of care to facilitating growth and healing in others.”

Being mindful in practice, the third sub-theme, is an important aspect of the TBRI® approach which raised potential challenges for participants as they *realised* the effects of their own personal histories and how these might impact their practice. This was explained by participants who related potential challenges as “personal triggers and reactions and the challenging nature of the behaviours”, “my own regulation and state - self-awareness and being able to connect with clients (adult/children) despite being triggered or having my own stress that I’m dealing with”. In such instances, having the capacity to self- regulate, or “managing self” as referred to by one participant, was identified as a challenge. One participant wrote “getting down to a child’s level and taking that step back so reflecting not responding to the child’s behaviour. That will be my challenge”. Here knowing how to *respond* was apparent but having the capacity to do so was considered a personal challenge. The impact of early life experiences, attachment styles and their influence on how adults function relationally is a critical consideration when working with children who come from adversity. Cassidy (2001) notes that before adults can begin to fully support others, they must attend to their own unresolved childhood or early adult histories. Thus, supporting those who work with children who have experienced trauma to become self-aware is an important aspect of any trauma-informed approach.

Within the theme of personal and professional capacity, the last sub-theme identified was changing beliefs. This concept was reflected in participant perceptions, as one participant identified their challenge as:

Letting go of ‘traditional’ responses to behaviours [for example] consequences that are a disconnected punishment rather than a natural consequence. I think I have started to break that stigma within myself since learning about TBRI [®] and all the aspects of it.

Whilst another noted “moderating my own response” and another “I think for myself [the strategy] compromises will be hard because I have grown up around the stigma that we have to do what we are told and if we don’t then there will be consequences”.

#### Time

The second theme that arose from participants’ perceptions of the challenges of TBRI® related to time. Here three sub-themes were identified, (i) time to interact with children, (ii) time to evidence change, and (iii) time to develop professional skills.

In sub-theme one, participants noted a significant barrier to implementing TBRI® in their context was the transient nature of the children and families with which they work. One participant noted “we work with children and families and mostly only have a snapshot in time. It is very hard to know and identify triggers”. Another reported that:

A barrier could be having the time to implement these strategies. Our families come into our centre for no longer than 1.5 h at a time [, and a] lack of consistent time with each child would be the biggest barrier for me personally. This makes it really hard to build a rapport with them.

One participant commented on the fact that some children may not come into their care until they are almost ready to start school and that support could no longer be offered once they moved to a different system of care. They noted:

Challenges include time frames; people grow and move so quickly and unfortunately our boundaries are quite clear with specific age groups. Once you have passed the age of 5 we can no longer offer the support that has been given. This will become a barrier especially if the family comes to our attention when the child is 4.5 and will only be within our company for 6-18months.

The second sub-theme was time to evidence change. This challenge was highlighted by a participant who stated:

It may take more than my allocated time with the family [and] the need to be seen to be ‘doing’ something… with ‘doing’ meaning quick outcomes from CBT style strategies. I don’t personally feel pressure from others to do this, but working with trauma impacted people is slow work that requires trust to be earned. It can feel like not doing anything when in truth deep relational work is happening.

This point is an important consideration as adding further pressure to an already challenging role has the potential to negatively impact wellbeing (Grant et al., 2019).

The last sub-theme, time to develop professional skills, was seen as a challenge. Here participants reported that time to develop and practice the strategies taught was difficult for them. One noted “the challenges are around providing the time and energy needed to support large groups of children with trauma particularly in the classroom”. Another reported that “making the framework become an integral part of everything we do - needs to become habitual. Lots of practice is needed at using the language and strategies”. This comment suggests the understanding of a shift from *doing* to *being* trauma-informed (Avery et al., 2022; Stephenson, 2023), a concept discussed above. It also articulates that this participant understood how to *respond* appropriately to *resist* re-traumatisation.

Throughout this theme of personal and professional capacity, the recognition of how to *respond* to children effectively was highlighted.

#### Continuity of Approach

The third theme that arose from participants’ perceptions of the challenges of TBRI® related to continuity of approach. Continuity was reported above as a positive of TBRI® and subsequently has also emerged as a challenge/barrier. Participants identified TBRI® as having the potential to offer a consistent approach, however, implementation was perceived to be a challenge. Having a consistent approach for children is well documented both within and across organisations (Colvin et al., 2021). This theme highlighted participants’ understanding of what is needed to *respond* to children in a trauma-informed way.

Here, participants shared their views around the need for a consistent approach from all those who cared for the child. One participant stated that we need to “clearly articulate the benefits [of TBRI®] for all people working with children and families of any age” whilst another noted a challenge as “other members of the team not understanding TBRI® and therefore creating a barrier to my newfound knowledge”.

This idea of a uniformed approach was not only considered for other professionals but was also identified as necessary for families. Here statements included “information and strategies need to be shared with the families to give children the consistency that is necessary to make changes” and that “enabling parents to parent in this manner” was necessary. The idea suggested by this participant that parents may need support to implement TBRI® was taken up by another participant who wrote:

Some of the parents of our children would not be/ or are unable to be supportive in our approach. Not all parents would continue the work we do at home in their own environment. Trauma may still be happening to some children. Parents can’t ‘deal with it’ when they are coping with their own effects of trauma.

Offering parents knowledge of trauma and responsive strategies for managing children’s behaviours may not be sufficient without more specialised family supports. The *recognition* of the impact of family histories and associated attachment issues identified by participants may, however, provide the understanding necessary to begin making small steps towards supporting families with a history of trauma. This concept was extended in the final theme discussed below.

#### Intergenerational Trauma

The final theme to emerge in relation to the challenges of TBRI® was related to the impact of intergenerational trauma. Here participants’ comments clearly articulated the context in which they work where many children come from families who are dealing with their own trauma histories. Specific challenges for participants were reported as “how we apply this [new knowledge] to inter-generational trauma situations”. As an example, this participant wrote:

I work with adults and children, so I’m trying to translate it to both the children and adults. I think the adults I work with carry significant trauma and are not at the developmental level one might expect of adults (particularly emotionally and socially), so I’m thinking how to use it with them.

Another participant noted a challenge as “working with families with parents and grandparents who are parenting through their own trauma” and “supporting parents who have a trauma history or are experiencing ongoing trauma to support their own children” and finally “I mostly work with adults, so thinking about how to apply the principles to adults who have the emotional maturity of children/teenagers [is a challenge].”

Participant responses here demonstrated knowledge in relation to *recognising* the signs and symptoms of trauma, *responding* in a trauma informed way, and *realising* how far-reaching this can be. The comments also highlighted the need to provide specific support for professionals working in such contexts. CFLCs were established to support families and their young children. This context, therefore, provides an ideal opportunity for professionals to support families who have experienced intergenerational trauma to heal and grow.

Overall, the findings from this study in relation to participant perceptions of TBRI® (RQ1) showed that TBRI® was relevant and contributed positively to participants’ work with young children and families. The TBRI® professional learning provided an opportunity for participants to deepen their knowledge of the impact of trauma and offered tools to support changes to their practice; all of which was presented within the context of, what was for some, previously held beliefs and concepts already familiar to them and their work.

Whilst this was a small-scale study, the findings align with similar studies undertaken to explore the effectiveness of approaches to supporting trauma informed practice in educational contexts. Several studies found that when the professional learning offered new knowledge that was relevant and relatable to participants’ work, this promoted a better understanding of the impact of trauma (Reid et al., 2018; Stipp & Kilpatrick, 2021; Sweetman, 2022; Thomas et al., 2019), increased participants’ empathy for children and families, and supported the development of relationships (Douglass et al., 2021). In addition, when strategies were offered that solved current problems participants were experiencing, changes to practice occurred (Cohen & Mehta, 2017).

Whilst challenges/barriers were reported, these related largely to the complexities inherent to working in vulnerable communities, rather than to TBRI® specifically. The notion of a transient population and pressures to find quick fix solutions to complex, long-term problems add to an already demanding role. Perhaps given these workplace constraints it is feasible that, at least initially, participants questioned their own efficacy. Douglass et al. (2021) reported an increase in confidence and empowerment for educators as their skill levels developed. This was echoed by Reid et al. (2018) who reported increased teacher confidence over time which enabled educators to create environmental change that provided trauma informed settings for both children and professionals working within them.

It is interesting to note that, whilst some participants reported a positive to be the alignment between new knowledge and previously held beliefs, for others, a misalignment was experienced and reported as a barrier to implementation. This challenge reminds us that professional learning intercepts a participants’ unique learning journey. This notion is worthy of consideration in further professional learning development since challenging previously held beliefs is critical if practice change is to occur but needs to be done through a reflective lens with thought, respect, and undertaken over time. Again, findings of Douglass et al. (2021) resonate, as their findings showed there was a need to challenge beliefs around relationships to enable practice change. The notion of self-reflection to support practitioners to better understand ‘who they are’, and how previous experiences and beliefs inform practice is central to addressing this challenge (Bobis et al., 2016; Brookfield, 2017; Door, 2014).

Finally, reported challenges such as time, continuity, and intergenerational trauma are reflective of broader social and systemic issues that can not easily be addressed by one approach in one context (Coates, 2017).

### The Effectiveness of TBRI ®

To explore the effectiveness of TBRI® as a trauma-informed approach (RQ2) from a theoretical perspective, the themes discussed above were analysed deductively to ascertain alignment with SAMHSA’s (2014) four Rs. Results of this analysis showed that TBRI® was effective for these participants as a trauma-informed approach since all four Rs aligned with the identified themes (see Table 1).

**Table 1:** *Alignment of positives and challenges/barriers of TBRI® to SAMSHA’s four Rs*

Positives	Realise	Recognise	Respond	Resist
Self-development	X	X	X	
Relationships			X	
Improved outcomes			X	
Evidence-based approach	X		X	X
**Challenges/barriers**				
Personal professional capacity	X	X	X	
Time			X	X
Continuity of approach		X	X	
Intergenerational trauma	X	X	X	

As can be seen in the thematic discussion above (see words written in italics) all four Rs were indicated in participant responses to the positives and challenges/barriers of TBRI® as an approach. Participant responses to the positives of the approach showed clear alignment with all four Rs as participants commented, not only on their increased *realisation* of the impact of trauma and how the approach had helped them to *recognise* symptoms of trauma but also how they had changed their practice to *respond* to children and families in a way that would *resist* re-traumatisation and support healing and development. Whilst the challenges/barriers showed alignment with participant understanding of the four Rs, some participants acknowledged that they faced challenges that potentially hindered their capacity to implement the approach effectively. Despite this barrier, the data evidenced participants’ understanding and knowledge in relation to the four assumptions.

Overall, an alignment of participant perceptions of TBRI® to SAMHSA’s four Rs revealed, within the context of two Tasmanian CFLCs that TBRI® was an effective trauma-informed approach. It is especially worth noting that whilst all four Rs were represented across the eight themes, the *respond* by putting theory into practice assumption aligned with all themes. This is again reflected in previous studies where participants identified the need for greater understanding along with appropriate strategies to enable them to better support the children in their care (Cohen & Mehta, 2017; Gorski, 2020; Reid et al., 2018; Stipp & Kilpatrick, 2021). This is a significant finding since the response of a professional to a child’s behaviours and needs has the potential to support growth and development or induce a fear response evoking challenging behaviours and possibly re-traumatisation of the child (Bath, 2015; Porges, 2017; Purvis, 2013).

### The Sustainability of Changes to Practice

To obtain an understanding of the sustainability of changes to practice (RQ3), participants were surveyed at three months and again at six months post professional learning. In the survey they were asked to report on whether they had used TBRI® in their work with children and families and if so, what their experiences had been.

At three months, eleven participants responded to the survey with nine of those reporting they had used TBRI® in their work. They reported that they had implemented all the TBRI® principles and as a result had added strategies to their practice and gained more confidence in implementation. This is summed up by one participant who wrote “[I] am feeling like I have added some new skills to my toolkit that help me connect and support children but in particular I have gained more confidence.” Participants noted how mindfulness was a skill that had been a focus, with one participant stating, “I am practicing using the right tools for challenging behaviour/ staying calm and listening more often!!” whilst another reported:

Putting myself in their shoes to use empathy and understand more of how they are feeling!! Offering support and understanding does help the children calm and lets them respond when ready to tell me what they need… and using less words helps them to regulate!

Whilst another indicated “I have tried to be more mindful of children’s hydration, warmth etc. and work with parents to support this” and finally, “I am conscious of the need to create a safe place in groups I run, and I offer empathy and acceptance to parents.”

Working with parents also seemed to have been a focus. This is not surprising as it was identified as a challenge in the earlier survey. Here using modelling to teach parents TBRI® strategies and support a more consistent approach for the child was reported. Participants stated they were:

Talking to parents a lot more about giving children warnings, a sign, a visual, a timer to help them transition between events [and] supporting parents to use phrases like ‘It is not OK to hit’ rather than ‘stop being naughty’ and then helping them to support their child in finding alternative strategies to meet needs.

One participant indicated:

As a team [we] have been trying to model a lot of playful interaction, naming up children’s initiatives and valuing them, and using behavioural matching. Helping parents to understand how to give a choice and still have a win for them but to give some control to their child.

Finally, a change of language across the centre was reported, with the life value terms (short phrases to support behaviours) being identified as useful.

At six months, survey responses had reduced to eight with seven of the eight reporting that they were still using TBRI®. The participants reported that, in addition to continuing to use familiar strategies such as “choices”, they had begun to explore other strategies and their confidence had continued to increase as these became more embedded into practice. Being mindful of how participants were interacting with children was evident in their responses, summed up by one participant who wrote “[I’m] much more focussed on ensuring the physiological needs are met… getting better at being a stress detective and looking for external factors that may be influencing the child and working out strategies to support them”. Finally, one participant summed up their experiences of using TBRI®:

Yes, I have had much success with these principles and continuing to be persistent and not giving up, it can be a process, but the rewards are wonderful to see. Now working in a school with kinder aged children all these strategies can be applied every day.

This study explored participant perceptions in relation to sustainability of changes to practice (RQ3) over a six-month period. Three and six months post the initial workshops, responding participants reported changes to practice and to the language they used. They stated they were using all the TBRI® principles. More specifically, they had added strategies to their practice, particularly mindful awareness, which is a core competency of TBRI® and identified after the initial workshops as a challenge for some participants. They also reported having extended their skills to include parental support, again something they reported was needed immediately post initial workshops. Finally, the increase in their confidence was evident from their responses. For these participants, changes to practice were sustainable and had permeated other aspects of work.

Cohen and Mehta (2017) discovered elements from professional learning that contributed to lasting reform. Many of these elements were also identified within the findings of the study reported in this paper. Whilst these findings offered promise regarding the sustainability of practice change over a six-month period, the response rate to the surveys at both three and six months were low. There are many reasons why surveys may not have been completed. The complex, busy and exhausting role undertaken by these participants would place reading and responding to emails low on their priority list; the completion of a survey would likely be even lower. An alternative reason maybe that participants were unable to overcome the challenges/barriers encountered and were no longer implementing TBRI® in their practice. Therefore, these initial findings warrant further exploration.

### Limitations and Recommendations of the Study

The main limitation of this study was population size. In addition to small numbers generally, researchers were also unable to differentiate perceptions according to professionals’ role. The requirement to ensure participant anonymity was also an imperative. Whilst participants’ reports were positive and aligned with SAMHSA’s four Rs, findings cannot be generalised. The researchers also encountered a decrease in the completion of surveys over the six-month period which further compounded the impact of having an already limited data pool. Thus, it is recommended that further studies involving CFLCs would increase the insight gained from this study. Expanding the research beyond CFLCs to other early childhood contexts, pre-kindergarten and kindergarten, would also inform current knowledge and increase understandings of TBRI® as a trauma-informed approach in the early childhood sector.

Other recommendations from this study identify the need to develop and implement parent workshops to increase continuity and support consistency between home and centre. Furthermore, it is proposed that time allocated to professional learning be extended to enable professionals to develop their new skills, overcome identified challenges/barriers within their context and subsequently make changes to practice. This additional time would also allow for additional support to increase practitioner confidence. Finally, it is recommended that strategies for working with all parents, including those with trauma histories be considered in future professional learning.

## Conclusion

This Tasmanian study explored the effectiveness of TBRI® in the early childhood context and from the perspective of the early childhood professional. In so doing, it responded to the “underexamined” perceptions of educators (Thomas et al., 2019, p. 422) and the lack of evidence base currently experienced around trauma-informed approaches in the education and care context.

The findings reported here provide a starting point for early childhood professionals who are seeking an evidenced based approach to support them in their work. Although findings from this study cannot be generalised due to its small scale, they do align with similar studies undertaken in the US (Reid et al., 2018; Stipp & Kilpatrick, 2021).

Participants documented many positives of the TBRI® approach including their self-development, relationships, improved outcomes, and an evidence base. Several challenges to implementing TBRI® were also identified. These included personal and professional capacity, time, continuity of approach, and intergenerational trauma. Each of these identified themes aligned with SAMHSA’s (2014) four Rs, recognised as essential components of any trauma-informed approach.

It is of significance that most of the challenges reported were more closely related to the application of TBRI® within the given context rather than to challenges inherent in the approach itself. Such challenges still need to be addressed, however, if the approach is to be effective for all early childhood professionals. The lack of self-confidence reported by some participants, particularly early in the professional learning, suggests it would be advantageous to offer practitioners more ongoing support and time to practice their new skills.

In addition, findings highlighting the challenge of intergenerational trauma cannot be underestimated. The need to focus on the family as a whole and not just the child is an important philosophical underpinning of CFLCs. Participants reported they were challenged to meet broader family needs specifically where intergenerational trauma was a contributing factor. Therefore, further exploration of how TBRI® might be used to meet the needs of family members with trauma histories is recommended. Since the TBRI® professional learning focused primarily on working with children, skills for working with adults were not a part of the workshops. This statement indicates another way in which the TBRI® approach might be extended to support this population.

As mentioned above, this was a small-scale study and the survey response rate declined over time. Participants who did respond at three and six months, however, reported an increase in confidence and sophistication of practice. These findings show promise relative to the effectiveness of TBRI® in the longer term for some participants. For others, however, providing the extra time and support suggested above may be necessary to sustain implementation and increase practice change.
